# Effectiveness of Line application and telephone-based counseling to improve medication adherence: A randomized control trial study among uncontrolled type 2 diabetes patients

**DOI:** 10.34172/hpp.2021.55

**Published:** 2021-12-19

**Authors:** Jukkrit Wungrath, Nattapong Autorn

**Affiliations:** ^1^Faculty of Public Health, Chiang Mai University, Chiang Mai 50200, Thailand; ^2^Doi Saket Hospital, Chiang Mai 50220, Thailand

**Keywords:** Medication adherence, Diabetes mellitus, Counseling

## Abstract

**Background:** More than 4.2 million cases of diabetes mellitus (DM) were reported in Thailand during 2019. Medication adherence is necessary to delay disease progression and prevent complications among uncontrolled type 2 DM patients. The objective of this research was to study how education via the Line application and telephone-based counseling impacted medication adherence knowledge by analyzing the behavior of uncontrolled type 2 diabetic patients.

**Methods:** Uncontrolled type 2 DM patients in Doi Saket Hospital, Doi Saket district, Chiang Mai province, Thailand. were included in the study. The sample was divided into an experimental (n=30) and control group (n=30). Patients who met the inclusion criteria of having uncontrolled type 2 diabetes diagnosed by a physician for at least one year, capable of communicating in Thai, possessing a mobile phone with the Line application and able to partake in activities for eight weeks were recruited in the parallel-group randomized trial. The experimental group participated in the developed education program, while the control group received standard routine health education activities provided by their health care providers. The intervention was based on the 5Rs principle as right medicine, right dose, right route, right patient and right time and included activities via the Line application and telephone-based counseling. Participants were evaluated for their medication adherence knowledge and behavior.

**Results:** After eight weeks of education through the Line application and telephone-based counseling, posttest mean scores of medication adherence knowledge of the experimental and control groups were 18.03 (SD=0.28) and 12.37 (SD=0.62), while posttest mean scores of medication adherence behavior of the experimental and control groups were 49.28 (SD=3.77) and 33.84 (SD=3.81), respectively. Results revealed that the experimental group had statistically significant (*P* <0.01) higher medication adherence knowledge and behavior mean scores.

**Conclusion:** Education using the Line application and telephone-based counseling program improved medication adherence knowledge and behavior among uncontrolled type 2 DM patients. Other outcomes of social media interactions such as patient engagement, patient behavior and attitudes, and the efficacy of patient-health care provider communication levels are possible areas for future study.

## Introduction

Diabetes mellitus (DM) is a chronic metabolic condition characterized by elevated blood glucose levels due to insufficient or ineffective utilization of insulin by the body.^1^ In adults, insulin resistance is the most frequent feature of type 2 DM.^1^The global prevalence of DM was 9.3% in 2019 and is forecast to climb to 10.2% by 2030,^2^ with increases in DM numbers halted by 2025.^2^ Over 4.2 million cases of DM were reported in Thailand during 2019.^3^ DM affects around 8.3% of the Thai adult population aged 20 to 79 years,^3^ and accounts for approximately 4% of total deaths by noncommunicable diseases (NCDs). An estimated 1.3 million Thai adults will live with DM by the end of 2035.^3,4^ Two-thirds of type 2 DM patients reside in cities.^3^ DM prevalence in urban areas is related to several risk factors including sedentary lifestyle, unhealthy diet and lack of time for regular exercise.^3,5^ Chiang Mai, the largest province in the north of Thailand, has seen dramatic growth in NCDs over the past two decades.^4^ A report by the Chiang Mai Provincial Public Health Office in 2020 stated that in Doi Saket district, Chiang Mai province, 292 type 2 DM patients were screened. They were all unable to control their blood glucose levels, while 112 patients (38.36%) were able to control their blood pressure levels. The first and second screening examinations revealed that 132 patients were unable to control their blood sugar levels, accounting for 45.20% of the total.^6^

Medication adherence is necessary to delay disease progression and prevent complications among uncontrolled type 2 DM patients.^3,7^ In adult patients with type 2 DM, a glycemic control goal of 7% HbA1c is advised,^8^ Poor glycemic control causes serious acute and chronic complications due to untreated or insufficient medication adherence.^8,9^ Related findings showed that proper glycemic control by patients who practiced medication adherence delayed disease progression, avoided the risk of complications, reduced death, prevented hospital admissions, reduced treatment cost and improved their quality of life.^10-12^ Knowledge and behavior were factors impacting medication adherence among patients with type 2 DM.^13^ Proper health education plays a vital role by supporting medication adherence and helps patients to overcome diabetic complications.^14-16^ Promoting appropriate health knowledge and behavior is, thus, a significant way to improve health by lowering the possibility of DM complications, increasing longevity and improving the quality of life.^17^ Most education programs employ teaching or training approaches. However, programs to elaborate knowledge and behavior among uncontrolled type 2 DM patients are lacking. Recently, Line applications and telephone-based counseling have been established to assist health promotion behavior. Previous research found that Thai people, including patients living in communities, used the Line applications and Facebook as the most popular social media platforms.^18,19^ The Line application is mostly used to chat with coworkers, family and friends, as well as to share and search for various health-related information.^20,21^ Nowadays, social media applications play significant roles in the daily lives of type 2 DM patients.

Patients with type 2 DM and health education support from health care practitioners using traditional approaches in communities have been the subjects of related studies by hospitals and DM clinics in rural areas of Thailand. However, lack of sufficient information exists regarding the role of health education using social networking, especially the Line application together with telephone-based counseling support in medication adherence among uncontrolled type 2 DM patients living in the community. Social networking using the Line application and telephone-based counseling to share information and follow up on medication adherence was investigated in this study. Findings can be used to promote health education via the Line application and telephone-based counseling to improve medication adherence habits among glycemic uncontrolled patients with type 2 DM living in Thai communities.

## Materials and Methods

A randomized control trial design was adopted, with participants recruited from the Doi Saket Hospital outpatient department in the Doi Saket district of Chiang Mai, Thailand. This research was conducted between October 2019 and January 2020.

### Participants

The study participants were uncontrolled type 2 DM patients at Doi Saket Hospital, Doi Saket district, Chiang Mai, Thailand. G*Power software was used to determine the sample size with a power of 0.80, alpha of 0.05 and an effect size of 0.80. The sample size per group was calculated at 26. An extra 10% was added to allow for losses, resulting in 30 samples in each group. Patients who met the inclusion criteria of having uncontrolled type 2 diabetes diagnosed by a physician for at least one year, capable of communicating in Thai, possessing a mobile phone with the Line application and able to partake in activities for eight weeks were recruited.

All subjects that satisfied the criteria were assigned randomly to the experimental or control groups. The purpose, actions, and procedures of the study were notified to all subjects who were free to withdraw at any time. Before starting the study, all subjects gave informed consent. All data collected during the study remained confidential.

### Procedure

*Experimental group*

*Line group activities*: The researcher presented eight video clips. Each had 5-7 minutes of content related to the 5Rs principle, namely, the right medicine, right dose, right route, right patient and right time. All the video clips were created by the researcher after conducting a literature review. The validity of each component was checked by the three experts. The video clips were sent through the Line application during the 2nd to 7th weeks at intervals of once a week on a Monday. Participants watched the video clips whenever they liked because all the clips were saved in the Line Notes application.

*Telephone-based counseling*: During the 3rd, 5th and 7th weeks of the program 5-10 minute counseling sessions were scheduled with the researchers. The participants were instructed through telephone-based counseling. This included activities to assist in reducing the chance of prescription errors. The participants were also given the phone number of the researcher and encouraged to get in contact if they required supplementary support regarding medication adherence issues.

Following the intervention, all participants received a standard routine program from their health care providers.

*Control group*

The customary routine and educational health care activities were received by participants in the control group from health care specialists in their communities. The control group did not receive eight weeks of education provided to the experimental group as the Line application and telephone-based counseling platform.

After completion of the experimental group, the intervention platform and the collection of data, the researchers administered the same intervention to members of the control group.

### Measurements

The questionnaire was split into three sections that were determined from the literature review.

*Part 1*: Age, gender, educational level, marital status, employment, family income and number of family members comprised demographic data collected from the samples.

*Part 2*: Diabetes Medication Adherence Knowledge (DMAK). The DMAK was created after a thorough assessment of the relevant literature and textbooks. The DMAK is a 15-item true-or-false questionnaire that was designed to assess patients’ knowledge of DM medication adherence. Each question answered correctly scored one point. Example questions included “Could diabetes drugs be discontinued when blood glucose level returns to a normal range?”, and “What are the side effects of diabetes drugs?”. The higher the total score, the better the patient’s knowledge. The validity of the DMAK was verified by three experts. The item objective congruence (IOC) index was 1.0 and Cronbach’s alpha was 0.80. Scores were divided as 0-5, 6-10 and 11-15 referring to low, moderate and good levels of knowledge, respectively.

*Part 3*: Diabetes Medication Adherence Behavior (DMAB). The DMAB comprises 15 questions with a three-rating scale (regularly practice, sometimes practice and never practice). For example, “Do you ever forget to take your diabetes drugs?” and “When you feel better, do you stop taking your diabetes drugs?”. The DMAB was assessed by three experts for content validity. The IOC index for all items was greater than 0.67 and Cronbach’s alpha was 0.88. Behavior levels were divided as 15-25, 26-35 and 36-45 referring to low, moderate and high levels of behavior, respectively.

### Data analysis 

The data were cleaned, checked for completeness and analyzed using STATA version 16 software. The Kolmogorov-Smirnov test was conducted to assess data normality. Descriptive demographic statistics were computed as frequencies and percentages for categorical variables and means and standard deviations for metric variables. Differences in demographic variables and study variables between the experimental and control groups at pretest were analyzed using the chi-square test for categorical variables and Fisher’s exact test for metric variables. The independent samples *t* test was used to examine differences between the experimental and control groups at baseline, while the paired samples *t* test was used to examine differences within groups before and after the intervention. The significance level (α) was set at *P*< 0.05.

## Results

### Participant demographics

In the experimental group over half the subjects were female (56.70%), with mean age 61.57 (SD = 11.23), married (70.00%), primary school graduates (83.30%), employed (60.00%), number of family members between 3 and 5 (56.70%) and average family income >10,000 baht/month (76.70%).

In the control group, most of the samples were female (66.70%) with mean age 59.17 (SD = 13.52), married (73.30%), primary school graduates (73.30%), employed (56.67%), number of family members between 3 and 5 (63.30%) and average family income >10 000 baht/month (63.30%) (Table 1).

**Table 1:** Subject demographics

**Demographic data**	**Experimental group** **(n=30)**		**Control group** **(n=30)**		***P*** ** value**
	**Number**	**%**	**Number**	**%**	
Gender					0.43^a^
Male	13	43.30	10	33.30	
Female	17	56.70	20	66.70	
Age (year)					0.97^a^
< 41	1	3.33	2	6.70	
41-50	4	13.33	6	20.00	
51-60	7	23.33	6	20.00	
61-70	13	43.33	12	40.00	
71-80	3	10.00	3	10.00	
>80	2	6.67	1	3.33	
Marital status					0.84^b^
Single	2	6.67	3	10.00	
Married	21	70.00	22	73.33	
Divorced/Separated	7	23.33	5	16.67	
Education level					0.67^b^
Primary school	25	83.33	22	73.33	
High school	3	10.00	4	13.33	
Vocational/University	2	6.70	4	13.33	
Occupation					0.79^b^
Employed	18	60.00	17	56.67	
Unemployed	12	40.00	13	43.33	
Number of family members				0.40^b^
< 3	7	23.30	9	30.00	
3 - 5	17	56.70	19	63.30	
> 5	6	20.00	2	6.70	
Average family income (Baht/month)			0.55^b^
< 5000	5	16.70	7	23.30	
5001-10000	2	6.70	4	13.30	
>10000	23	76.70	19	63.30	

SD, Standard deviation. ^a^ Fisher’s exact test (*P* value < 0.05), ^b^Chi-square test (*P* value < 0.05).

There were no important differences between the two groups at baseline (*P*= 0.43), implying that demographic data of the experimental and control groups were similar.

### Effectiveness of the program on study outcomes 

No statistically significant difference was found in pretest medication adherence knowledge scores between the two groups (*P*= 0.26), as indicated in Table 2. Experimental group scores averaged 18.03 after the intervention, while the control group averaged 12.37. The difference between the two groups was statistically significant (*P* < 0.001). A statistically significant change was also recorded in the experimental group scores before and after the intervention (*P* < 0.001) but no significant difference was found between the control and experimental groups (*P* = 0.68).

**Table 2:** Independent and paired samples *t* tests results for medication adherence knowledge between the two groups

**Medication adherence knowledg**e	**Experimental group**		**Control group**		***P*** **value** ^a^
	**Mean**	**SD**	**Mean**	**SD**	
Before	13.33	0.65	12.27	0.68	0.26
After	18.03	0.28	12.37	0.62	<0.001*
*P*value^b^	0.00*		0.68		

*P < 0.05; ^a^ Independent samples *t* test; ^b^ Paired samples *t* test.

There was no statistically significant difference in pretest medication adherence behavior scores between the two groups (*P* = 0.88), as indicated in Table 3. Experimental group scores averaged 49.28 after the intervention, while control group scores averaged 33.84. The difference between the two groups was statistically significant (*P* < 0.001). There was also a significant change in the experimental group scores before and after the intervention (*P* < 0.001) but no significant difference was recorded in the control group (*P* = 0.33).

**Table 3:** Independent and paired samples *t* tests results for medication adherence behavior between the two groups

**Medication adherence behavior**	**Experimental group**		**Control group**		***P*** **value** ^a^
	**Mean**	**SD**	**Mean**	**SD**	
Before	32.78	4.65	33.16	3.48	0.88
After	49.28	3.77	33.84	3.81	<0.001*
*P* value^b^	0.00*		0.33		

*P < 0.05; ^a^ Independent samples *t* test; ^b^ Paired samples *t* test.

## Discussion

Significant positive changes were recorded from the baseline of all study outcome variables among participants in the experimental group compared with the control group after evaluations during the eight weeks of study. Results indicated that education using the Line application and telephone-based counseling programs was effective. Participants subjected to the medication adherence education program had significantly higher mean scores on medication adherence knowledge and behavior than those who received ordinary health care and conducted routine health education activities. The data also confirmed the positive impacts of employing the Line application and telephone-based counseling program for medication adherence instruction. This increase in diabetes medication adherence knowledge and behavior was consistent with related studies.^22-24^ Findings emphasized the importance of providing specific information and counseling.

The general public, patients and health professionals utilize social media, especially the Line application in health care for a variety of reasons to gain numerous benefits.^25-27^ In Thailand, Maneenithiveth^28^ discovered that a self-care program delivered via the Line application effectively improved health literacy and clinical parameters, with blood glucose and cholesterol levels significantly decreased in the experimental group. Wannasiri et al^29^ assessed the use of the Line application for diabetic retinopathy prevention on clinical outcomes in diabetic patients in Thailand. Results revealed that after program implementation for three months, mean scores of knowledge and self-care behaviors for diabetic retinopathy significantly increased. They suggested that this educational program should be used for patients in diabetic clinics to help delay the onset of diabetic retinopathy. Similarly, Chanon^30^ developed a model to promote self-care for patients with DM. The intervention group participated in activities via the Line application and telephone follow-ups for 15 days. When comparing pre- and post-intervention results, DM patients in the intervention group had considerably greater knowledge and self-care behavior, with lower blood sugar levels compared to the control group. Chanon recommended adopting this model in other hospitals to better care for DM patients. Likewise, Saibuathong^31^ concluded that the Line application was a good social media platform for instructing diabetic patients. A video clip sent via the Line application was used to educate patients. Results showed that the patients appreciated being able to educate themselves using online technology.

Telephone-based counseling proved effective in increasing medication adherence in this study. The effectiveness of telephone-based support for metabolic control of older adult patients with DM was also investigated by Becker et al.^32^ They found that telephone-based counseling was an effective method to deliver medical education to diabetic older adult patients and resulted in reduction of fasting blood glucose levels. In combination with other treatments, this may help to lower glycated hemoglobin levels. Furthermore, Mochari-Greenberger et al^33^ investigated the impact of tele-behavioral therapy on the self-management of DM patients. The intervention group showed a significant reduction in mean glucose levels. Participants in a DM behavioral tele-health program recorded significant reductions in glucose levels and increased frequency of glucose self-testing. Significantly increased medication adherence knowledge and behavior in our study corresponded to related studies using telephone-based counseling. This method is more practical for community health centers than clinic-based models. Duncan^34^ reported the effectiveness of telephone health education on promoting knowledge and safe practices among older adults with chronic illnesses. One advantage of telephone-based counseling is that participant engagement and usage can be monitored throughout the intervention period as an educational delivery mode for health promotion. Patients who received diabetic instruction and counseling over the phone had better clinical results than those subjected to the traditional approach.^35-37^

Findings of this study demonstrated that the Line application and telephone-based counseling were effective for educating self-care in uncontrolled type 2 DM patients. These widely accepted informatics systems increased the intensity and breadth of information provided to patients concerning medication adherence.

## Conclusion

Our study provides evidence that integrating the Line application with telephone-based counseling improved the self-care knowledge and behavior of medication adherence among uncontrolled type 2 DM patients. The use of these interventions was associated with positive patient outcomes. Public health sectors and other health care providers who strive to improve the self-help medical knowledge of diabetes patients should consider using social media channels as intervention applications.

The intervention was conducted in a community where most participants had low socioeconomic status and level of education. Therefore, the study outcomes may not be representative of the general population. Our findings can be generalized only to individuals with similar demographics to the study subjects. Assessing outcome variables within eight weeks is also a short timeframe that may not reflect the persistence of effective medication adherence. Future research should explore the intervention impact on medication adherence knowledge and behavior among uncontrolled type 2 DM patients by extending the length of the observation period and examining the effects of intervention in diverse population groups.

## Recommendations

In order for this intervention to be effective, healthcare providers need to improve their knowledge of how to provide information through mobile interventions to patients. Healthcare organizations need to develop related guidelines to support the use of Line application and Telephone-based counseling as health interventions. Organizations also need to provide related programs or training to provide accurate information and thus effective and efficient care to patients.

## Acknowledgements

The authors gratefully acknowledge the data input provided by the Faculty of Public Health, Chiang Mai University, and Doi Saket Hospital, Chiang Mai province, Thailand. The cooperation of all participants during this study was also highly appreciated.

## Funding

No funding was received from any institution or department.

## Competing interests

None declared.

## Ethical approval

The Ethics Research Committee of the Faculty of Public Health, Chiang Mai University approved this study (approval code: ET009/2018).

## Authors’ contributions

JW reviewed the literature, designed the research, collected and analyzed the data and approved the final manuscript. NA collected and analyzed the data, and also approved the final manuscript.
